# Questioning the evidence for BCI-based communication in the complete locked-in state

**DOI:** 10.1371/journal.pbio.2004750

**Published:** 2019-04-08

**Authors:** Martin Spüler

**Affiliations:** Department of Computer Engineering, Eberhard-Karls University Tübingen, Tübingen, Germany; Charité—Universitätsmedizin Berlin, Germany

When Birbaumer and colleagues [1] showed in 1999 for the first time that a person in a locked-in state can use a brain–computer interface (BCI) to communicate, it also created the hope of BCIs restoring communication in the complete locked-in state (CLIS), in which a patient has no remaining muscle control. Since this pioneering work, multiple electroencephalography (EEG)-based BCI systems have been successfully tested with locked-in patients [2]. However, these systems did not work for completely locked-in patients, leading to the conclusion that voluntary brain regulation is not possible in the CLIS [2,3].

This changed in 2014 when Gallegos-Ayala and colleagues [4] presented a case study suggesting that near-infrared spectroscopy (NIRS) could be used for communication in the CLIS. It was followed up by Chaudhary and colleagues in 2017 [5], who recorded NIRS in 4 patients in the CLIS. In that work, results from offline and online classification are presented with accuracies significantly above chance level, which led the authors to the conclusion that NIRS-based BCI communication is working in CLIS.

For this commentary, I performed a reanalysis of the data from Chaudhary and colleagues [5]. As the results are substantially different from the results reported in the original paper, I question the claim of NIRS-based BCI communication in the CLIS.

## Reanalysis of NIRS data

For the reanalysis, the data that were published as supplementary material [6–9] to the original paper were used. These data contain the preprocessed NIRS signal (HbO) for 20 channels and all trials of all training and feedback sessions, separated into "true/yes" and "false/no" trials. As Chaudhary and colleagues did not make available all data of the original paper, the number of sessions in this reanalysis may differ from the number in the original paper (for more details, see S1 Text).

### Statistics

Two different statistical analyses were performed, and both showed no significant difference in the NIRS response between yes/no questions. The first analysis was different than the one performed by Chaudhary and colleagues and is presented in S1 Text in more detail. For the second analysis, I point out methodological flaws in the statistical analysis of Chaudhary and colleagues and show results with corrected methods.

As the statistical analysis by Chaudhary and colleagues was not described with sufficient detail to exactly reproduce it, Chaudhary and colleagues responded in the first review round to this comment that they averaged the data first over all trials and then over all sessions and performed a *t* test on those averages. The problem with this kind of analysis is that the variance over trials/sessions is removed by the averaging, and only the variance over the channels is retained. Performing a statistical test will then compare the mean of yes-trials with the mean of no-trials while considering the variance over all channels. As the channels are highly correlated (not independent), the variance is very low and will lead to the wrong result, that the difference is significant. The statement that this kind of analysis is not correct can be tested by using a permutation test. Using a random permutation of the trials, instead of a separation by yes/no, should show no significant difference if the statistical analysis is correct. But for the method used by Chaudhary and colleagues, the results are significant in each of 10 random permutations (see figure A1 in S1 Text), which demonstrates that the used method is not correct.

**Fig 1:**
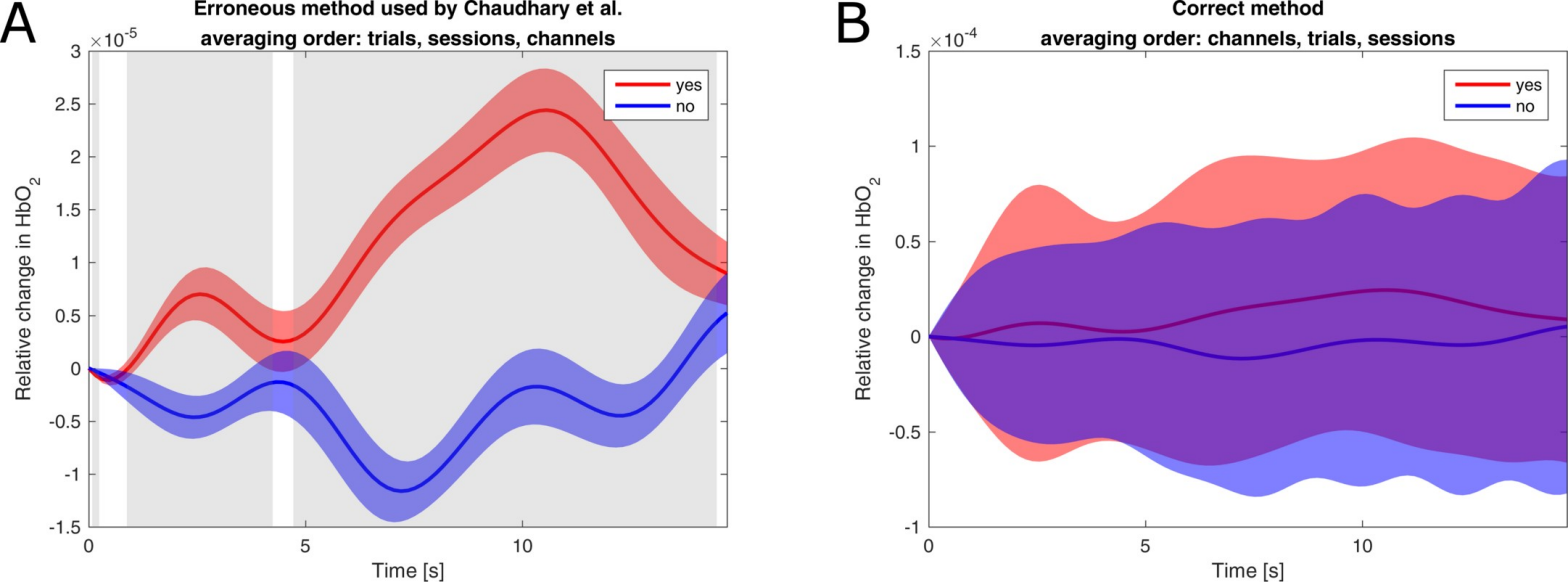
Comparison of statistical methods on data of patient B. Both plots show the average response to yes/no questions as well as the standard deviation. (A) Method used by Chaudhary and colleagues, first averaging over trials, then over sessions, and then over channels. For the *t* test, the variance over channels is retained and used. Time points with significant difference (*p* < 0.0005) are marked gray. (B) Same method but with a correct order of averaging, first averaging over channels, then over trials, and then over sessions. For the *t* test, the variance over sessions is retained and used. No time points show a significant difference (*p* > 0.05).

This does not mean that averages should not be used for statistical analysis, but that the order of averaging matters. If the data are first averaged over channels and then over trials, the variance over sessions is retained. But in this case, there is no significant difference between yes/no questions (*p* > 0.05; *t* test, not corrected for multiple comparisons) for any patient. Fig 1 shows the results of an analysis of data from patient B with the two different orders of averaging. Although the mean response is the same for both analyses, the variance and the results of a statistical test are different.

Regarding the averaging over channels, it should also be mentioned that averaging across channels is generally not recommended. Although it is not incorrect, averaging channels can reduce or even cancel out an effect in the data if the signal of interest is highly localized or shows different patterns across different brain areas. For this reason, an analysis treating each channel independently (as in S1 Text) is recommended.

### Offline classification

The offline classification of the data was also reproduced. As some details of this analysis were not given by Chaudhary and colleagues (e.g., hyperparameter), it was not possible to reproduce the results with exactly the same method, but a similar approach was used. A support vector machine (SVM) with a linear kernel was applied to the relative change in HbO${}_{2}$. For each day of each patient, a 10-fold cross-validation was performed in which the data were randomly divided into 10 blocks, and 9 blocks were used for training the classifier and tested on the remaining block. This process was repeated 10 times so that each block was used for testing once. For training the classifier, the training data were balanced by randomly removing trials of the majority class from the training data. The optimal hyperparameter C for the SVM was estimated by performing a grid search with a 10-fold cross-validation on the training data. When using the preprocessed data as input for classification, an average accuracy of 49.4% was obtained. The performance was not significantly different [10] than chance level (*p* > 0.05, not corrected for multiple comparisons) for all of the 42 days. More detailed results can be found in S1 Text.

## Discussion

In summary, a reanalysis of the data from Chaudhary and colleagues [5] has shown no significant difference in the hemodynamic response to "yes" and "no" questions, and the NIRS data could not be classified with an accuracy significantly above chance level. As the obtained results are in contrast to the results presented by Chaudhary and colleagues, possible reasons should be discussed. For the statistical part, it was possible to pinpoint the methodological flaw and therefore explain the erroneous results. Because of the lack of details in the description of the offline classification of Chaudhary and colleagues (e.g., choice of hyperparameter), one can only speculate, and it is up to Chaudhary and colleagues to provide more information and an explanation for why their offline results cannot be reproduced and how they achieved online accuracies above 70%.

As the data recorded by Chaudhary and colleagues show that their approach of a NIRS-based BCI does not work, it should be discussed if the current literature shows any evidence for communication in CLIS. The work of Gallegos-Ayala and colleagues [4] is the precursor study to the work by Chaudhary and colleagues [5], uses the same approach and the same patient (patient F), and therefore can also be doubted.

Regarding the use of EEG-based BCIs for communication in CLIS, Guger and colleagues [11] have recently claimed that their mindBEAGLE system, using a P300 paradigm, works also for CLIS patients. However, Guger and colleagues rest that claim on a very small sample size of 10 trials and do not provide any statistical assessment of their results. When performing a statistical analysis on that data, the classification accuracies are actually not significantly (*p* > 0.05) above chance level (see S1 Text for details).

While none of the papers provide sufficient evidence that CLIS patients can use a BCI to communicate, Chaudhary and colleagues [5] documented a shift of the dominant frequency in the EEG from the alpha band to the theta band, and a similar result was found by another group [12]. As the alpha rhythm is often used as a neurophysiological marker for consciousness or cognitive abilities [12], it raises the question if CLIS patients have an intact cognitive processing, as is generally assumed [5]. The slowing dominant frequency could rather indicate impaired cognitive abilities or an altered state of consciousness, which would explain why all attempts for BCI-based communication in the CLIS have failed so far.

Until the internal state of said patients is better assessed in future studies, it can only be concluded that there currently is no scientifically sound evidence that demonstrates communication in the complete locked-in state.

## Supporting information

S1 Text Appendix containing a more-detailed description of the methods and results of the reanalysis. (PDF)

S1 File Zip archive with scripts contains all Matlab scripts that were used for the statistical analysis, offline classification, and generating figures for this comment. (ZIP)

## Abbreviations

BCI: brain–computer interface
CLIS: complete locked-in state
EEG: electroencephalography
NIRS: near-infrared spectroscopy
SVM: support vector machine
